# Undergraduate Nursing Students’ Experiences of Individualized Digital Reminiscence Using the InspireD App in Care Home Placements: Qualitative Focus Group Study

**DOI:** 10.2196/91515

**Published:** 2026-05-19

**Authors:** Aoife Conway, Assumpta Ryan, Deirdre Harkin

**Affiliations:** 1 School of Nursing and Paramedic Science, Faculty of Life & Health Sciences Ulster University, Derry-Londonderry Campus Derry, Northern Ireland United Kingdom

**Keywords:** reminiscence, digital health, dementia, person-centered care, care homes, implementation, qualitative research, individualized, digital technology

## Abstract

**Background:**

Care home placements offer important opportunities for student nurses to develop relational and person-centered approaches to dementia care. Digital reminiscence platforms are increasingly used to support the well-being of people living with dementia; however, little is known about how such platforms may shape student learning within practice settings. There is limited qualitative evidence examining how digital reminiscence is experienced by students and how it influences their understanding of personhood, relationships, and care practices.

**Objective:**

The aim of the study is to explore undergraduate nursing students’ experiences of engaging with individualized digital reminiscence using the InspireD reminiscence app during care home placements.

**Methods:**

Following a pilot implementation of the intervention, a qualitative exploratory study was conducted, in which 13 undergraduate nursing students participated in 4 focus groups. Data were analyzed using reflexive thematic analysis.

**Results:**

Three themes were developed to capture how participants made sense of their learning and practice experiences when engaging with individual reminiscence using the InspireD reminiscence app. The first theme, “deepening empathy and understanding through reminiscence,” describes how participants developed a greater appreciation of residents’ life histories and personhood. The second theme, “learning through connection,” reflects how relationships with residents and families shaped communication, confidence, and emotional engagement. The third theme, “growing as person-centered practitioners within the realities of care home practice,” highlights how participants reflected on translating this learning into practice while navigating organizational constraints and everyday care demands.

**Conclusions:**

Findings suggest that the InspireD reminiscence app can support the development of person-centered learning within care home placements, although successful implementation is contingent on supportive organizational cultures. These findings contribute to wider discussions in health professions education by illustrating how digital platforms can mediate experiential learning in practice settings and support the preparation of future health professionals to use digital tools in relational and values-based ways. Future research should examine longer-term learning outcomes and implementation across diverse placement contexts.

## Introduction

### Background

Dementia is a leading cause of disability, dependency, and death among older adults, with nearly 10 million new diagnoses each year, and global prevalence continuing to rise [1]. In the United Kingdom, around 70% of care home residents have dementia or severe memory problems [2]. Given this high prevalence, preparing a workforce capable of delivering high-quality dementia care is essential. Previous work has emphasized that undergraduate nursing curricula must provide not only theoretical knowledge but also experiential learning that connects classroom teaching with clinical practice [3].

Care homes frequently support student nurses as clinical placement environments and provide important opportunities for developing competence. These settings represent distinct and complex learning contexts [4] where students meet, care for, and learn from residents with multifaceted health and social care needs. Person-centered practice is fundamental to high-quality dementia care in these settings [5,6]. Reminiscence contributes to this practice by supporting identity and fostering meaningful engagement [7,8]. Reminiscence is a well-established psychosocial approach that draws on personal memories to support communication and relationship building [9,10]. One-to-one reminiscence using individual memorabilia has strong evidence for enhancing communication and emotional connection [10]. Increasingly, digital apps provide platforms to collate meaningful memorabilia and create opportunities for reminiscence [11,12]. This study contributes to the literature by examining how the InspireD reminiscence app facilitates experiential, values-based learning in authentic practice settings.

The InspireD reminiscence app is a cross-platform tablet app developed to support personalized reminiscence through the selection, organization, and retrieval of multimedia life-story material. It was created using an Agile co-design process with people living with dementia and family caregivers in partnership with the Reminiscence Network Northern Ireland. The interface uses a simple stepwise structure and clear visual cues to minimize cognitive load and enable both shared and independent use. Users can upload and display meaningful content, including photographs, music, video clips, and recorded notes, with these individualized cues intended to stimulate memory, prompt conversation, and strengthen identity-focused engagement [12].

The InspireD reminiscence app was not designed as a tool for use in nursing education; however, it holds considerable potential for experiential learning, aligning with Kolb’s [13] theory that learning is strengthened through a cyclical process of engaging in experience, reflecting on that experience, developing new conceptual understandings, and applying these insights in future practice. This process also reflects broader constructivist principles in which students build meaningful understanding through authentic engagement with individuals and their lived experiences [14,15]. Several authors argue that higher education programs for nurses must align with contemporary educational priorities that emphasize digital literacy and aim to prepare practitioners to work confidently within digital health systems [16,17], supporting safe and effective technology use and meeting growing informatics demands [18]. Yet, the integration of digital health technologies within undergraduate nursing curricula remains inconsistent [19]. As health care systems continue to evolve, nursing students require structured opportunities to develop foundational competence in digital health technologies and to further consolidate these skills as they progress into professional practice.

The potential value of digital reminiscence platforms for supporting experiential learning among student nurses has received limited attention. Existing literature on digital reminiscence typically focuses on outcomes for people living with dementia and their familial caregivers [11,12,20] rather than on how such tools might facilitate student learning within care environments. Additionally, very little educational research or intervention work has been conducted in care home settings with student nurses, which further contributes to the novelty of this study [21]. To date, no studies have examined how student nurses engage with, and learn through, a digital reminiscence platform during care home placements. Addressing this gap provides an important opportunity to explore the educational potential of these technologies within complex practice environments [22].

### Aim

The aim of the study is to explore undergraduate nursing students’ experiences of engaging with individualized digital reminiscence using the InspireD reminiscence app during care home placements.

### Objectives

The objectives of this study are (1) to explore how engagement with the InspireD reminiscence app shaped students’ understanding of residents’ life histories, personhood, and dementia-related presentation; (2) to examine how engaging with digital reminiscence shaped students’ perceptions and approaches to person-centered care; and (3) to explore how students reflected on applying this learning in practice, including organizational challenges and constraints within care home settings.

## Methods

### Study Design

A qualitative exploratory study was conducted to explore undergraduate nursing students’ experiences of engaging with individualized digital reminiscence in care home placements following a pilot implementation.

### Ethical Considerations

Ethics approval was obtained from Ulster University Ethics Committee (reference: FCNUR-25-029-A). The study was conducted in accordance with the principles of the Declaration of Helsinki and relevant institutional governance procedures. All participants received an information sheet outlining the purpose of the study, what participation involved, and their right to withdraw without consequence. Written informed consent was obtained prior to data collection and reconfirmed verbally at the start of each focus group. To protect privacy and confidentiality, all data were anonymized prior to analysis. Potentially identifying information was removed, and participants were assigned unique codes. Participants were reminded at the beginning of each focus group to be mindful of confidentiality and asked not to share discussion content outside the group. Any quotations presented in the results are anonymized and reported in a way that prevents identification of individual participants or sites. Participants did not receive financial compensation for taking part in the study.

### Setting

The study took place in 7 care homes in Northern Ireland, all of which had existing partnerships with the research team through the My Home Life program, a leadership support program that aims to enhance the quality of life for all those who live, work, and visit care homes. These partnerships provided an appropriate context for exploring learning and practice development, as participating homes were already engaged in reflective practice, quality improvement, and student placement provision. The homes varied in size and geographic location (urban or rural) and in their prior exposure to digital technology in care delivery.

### Participants and Recruitment

Eligible participants were second-year student nurses undertaking a BSc (Hons) in adult nursing or mental health nursing at the participating university, allocated a placement in one of the participating care homes between June and August 2025, and willing to provide informed consent. Students who did not meet these criteria were excluded. Second-year nursing students were selected as they had completed the Level 2 Dementia Education Program, a mandatory component of the undergraduate curriculum at the participating university [23]. This program provides a theoretical foundation in person-centered dementia care and includes teaching on reminiscence principles along with an introductory classroom-based overview of the InspireD reminiscence app. This prior preparation was considered important for supporting experiential learning.

As this was a pilot intervention conducted within a defined cohort of students allocated to participating care homes during a single placement period, recruitment was necessarily bounded by the available population. All eligible students were invited to participate (n=18). Recruitment was conducted via email invitations and follow-up online information sessions to provide information on the study. In total, 13 students provided informed consent and took part in the focus groups. A total of 2 students declined due to competing academic or placement commitments, while the remaining students did not provide a reason for nonparticipation. Final sample size was judged to be adequate in relation to the focused study aim, the specificity of the sample, and the shared educational and placement context of participants [24].

### Intervention

All participating students were invited to attend a short orientation session delivered by the research team at the beginning of their placement. This session introduced the principles of reminiscence, demonstrated the InspireD reminiscence app, and addressed safeguarding, consent, and confidentiality in the context of practice learning. Each student received a dedicated iPad for use during the project. This remained in the care home following completion of the study. The app itself is publicly accessible and free to download.

Once on placement, students worked with care home staff to identify a suitable resident and to set up the app. The app enabled users to upload, organize, and present individual multimedia such as photographs, music, and video clips to support individualized reminiscence. Where possible, families were involved in sharing material and offering background information. The material uploaded to the app was determined by what residents or families wished to include. Decisions regarding which residents might benefit from engagement in reminiscence activity, including consideration of capacity and agreement to take part, were made by care home staff in accordance with their usual care planning processes and organizational policies. The reminiscence activity was undertaken as part of routine care and learning within the placement context. Care home staff remained responsible for monitoring residents’ well-being during reminiscence activity and followed usual care procedures if a resident became distressed. Residents were not recruited as research participants, and no resident-level data were collected. The research team did not access, moderate, or review any content, and no data were extracted directly from the app.

All students engaged in individualized reminiscence activity throughout their placement period (typically 6 weeks). All participants were involved in both developing and gathering personalized content and using this material in direct interaction with residents. Students were encouraged to focus on material that reflected the resident’s interests, relationships, or significant life events, in keeping with the principles of person-centered care. Use of the app was intentionally flexible. There was no set schedule or required number of interactions, and variation in how students integrated the app into their work was expected. Students were encouraged to introduce, adapt, and negotiate the use of the app when it felt appropriate and to adapt to residents’ preferences, communication abilities, and daily routines. The intention was to support authentic use of reminiscence within the constraints of everyday care home practice. The intervention is therefore understood as the process of engaging in individualized digital reminiscence within practice, rather than a fixed or standardized number of sessions; therefore, variation in exposure between students was expected and not controlled.

### Data Collection

Data were collected through focus groups (n=4) held at the university to ensure a neutral environment and minimize workplace influence on responses. Focus groups included between 2 and 5 participants (1 group with 5 participants, 2 groups with 3 participants, and 1 group with 2 participants) and lasted 42-60 minutes (mean duration 52, SD 7.5 minutes). Sessions were facilitated by an experienced qualitative researcher (AC), with a second researcher taking field notes (DH). Focus groups were conducted throughout August 2025.

Focus groups were selected to explore students’ subjective experiences within a shared placement context. The research team anticipated that collective discussion would stimulate reflective dialogue and enable participants to build on, contrast, and clarify each other’s accounts of their shared placement experiences. This approach is consistent with methodological literature that positions collective interaction as a means of eliciting perspectives, attitudes, and experiences that are shaped through group processes [25]. Given that participants had undertaken placements within dementia care settings, interaction was considered likely to deepen rather than dilute reflection. The focus group format also aligned with the pedagogical context, as students were accustomed to facilitated group reflection within their program.

The researcher who facilitated the focus groups was also involved in teaching within the program and had an existing professional relationship with participants. This dual role was recognized as having the potential to influence participation and disclosure; however, it also provided an in-depth understanding of the curriculum, placement context, and the pedagogical intent of the intervention, which supported a nuanced and contextually informed interpretation of the data. The potential for social desirability bias in focus groups with classmates, and in the context of a researcher and intervention affiliated with the same institution, was explicitly considered. To mitigate this, students were informed verbally and in writing that participation was voluntary, that they could withdraw at any time without consequence, and that nonparticipation would not affect their placement experience or academic progression. Emphasis was placed on the value of both positive and critical perspectives, and instances of divergent views were observed within the discussions. Focus groups were conducted in a neutral university setting to reduce the influence of the clinical placement environment. Reflexive field notes were maintained throughout data collection and analysis to critically examine how the researcher’s insider position and prior involvement in the program might shape interactions, interpretation, and theme development.

A semistructured focus group topic guide was used to maintain consistency while allowing participants to elaborate on their experiences. Demographic data were also collected. The guide was developed by the research team and informed by the study aims and objectives, the relevant literature on reminiscence and implementation in care home settings, and pedagogy in dementia. The guide was reviewed by the research team prior to data collection to ensure clarity, relevance, and appropriateness for the context. It was piloted with a small group of students who were not involved in data collection, and minor refinements were made to the wording and sequencing of questions.

All sessions were audio-recorded with consent and transcribed verbatim, and transcripts were anonymized.

### Data Analysis

Data were analyzed using Braun and Clarke’s [26] reflexive thematic analysis. Theme development was led by the primary analyst (AC), with additional members of the research team (AR and DH) contributing through reflexive dialogue and critical discussion to challenge interpretations and support interpretative depth and theme refinement. Analysis centered on developing patterns of shared meaning across the dataset rather than examining interactional dynamics within individual focus groups. Themes were generated as interpretative analytic outputs rather than descriptive summaries of participants’ accounts [26]. Coding was inductive, with themes developed iteratively and refined to ensure that they reflected the dataset [26]. Individual quotations are presented to illustrate how these patterns were articulated by participants. All researchers had prior experience in dementia care and digital health, which informed interpretation; reflexive notes were maintained throughout to interrogate assumptions and document analytic decision-making. Transcripts were stored and managed in Microsoft Word, with coding undertaken manually using document annotation functions (eg, comments and highlighting), with codes subsequently collated into thematic groupings using analytic memos. Transcripts or themes were not returned to participants for comment.

The study was conducted in line with the COREQ (Consolidated Criteria for Reporting Qualitative Research) [27] and the iCHECK-DH (Guidelines and Checklist for the Reporting on Digital Health Implementations) [28] to support transparent and comprehensive reporting of the qualitative methods and the digital health implementation components.

## Results

### Overview

In total, 13 second-year student nurses participated in the project and provided informed consent to take part in the focus groups. Demographic details are provided in Table 1.

**Table 1:** Participant characteristics of second-year nursing students who participated in focus groups following a pilot implementation of the InspireD reminiscence app in care home placements (N=13).

Categories		Participants, n (%)
**Course of study**		
	Adult nursing	7 (54)
	Mental health nursing	6 (46)
**Age group (years)**		
	18-25	3 (23)
	26-35	5 (38)
	36-45	3 (23)
	46-55	2 (15)
**Sex**		
	Male (including trans men)	2 (15)
	Female (including trans women)	11 (85)
**Ethnic group**		
	Black, Black British, Caribbean, or African	2 (15)
	White	10 (77)
	Mixed or multiple ethnic groups	1 (8)
**Informal caregiving experience**		
	Yes	7 (54)
	No	6 (46)
**Formal caregiving experience**		
	Yes	9 (69)
	No	4 (31)

Three themes were developed to capture how participants made sense of their learning and practice experiences when engaging with individual reminiscence using the InspireD reminiscence app. The first theme, “deepening empathy and understanding through reminiscence,” describes how participants developed a greater appreciation of residents’ life histories and personhood. The second theme, “learning through connection,” reflects how relationships with residents and families shaped communication, confidence, and emotional engagement. The third theme, “growing as person-centered practitioners within care home realities,” highlights how participants reflected on translating this learning into practice while navigating organizational constraints and everyday care demands.

### Theme 1: Deepening Empathy and Understanding Through Reminiscence

Students described how accessing meaningful memorabilia through the app shifted how they perceived the individuals they were supporting, encouraging a focus on the person rather than solely on the diagnosis of dementia:

She just completely opened then and loved telling me stories. I see her differently now. I feel like I actually see her now.

Participant 3, focus group 1

Students came to recognize the importance of understanding “a life lived” (Participant 2), developing deeper empathy and respect as they encountered residents’ histories:

There is definitely a whole life behind this person that they’ve lived, they’re in their 90s so they’ve lived a lot longer than we did and at different times ... it’s so beautiful what they achieved and done, you have to respect that.

Participant 6, focus group 2

Students described how using the app enabled them to see aspects of residents that were often missed in routine care. For many participants, this recognition signaled a shift in how they understood the resident, and for some, this provided a sense of purpose and motivation to identify ways of engaging with memory and personhood, reinforcing the idea that meaningful action remained possible despite the person’s current presentation: “I suppose there’s always ways of restoring memories in some way like, something you can do” (Participant 9, focus group 3).

The students discussed witnessing changes in mood, interaction, and communication when residents engaged with individual reminiscence. Students described a sense of “awe” (Participant 2, focus group 1), as previously withdrawn residents became animated when presented with individually meaningful cues:

I think what really stood out for me was ... I’d been there 9 weeks together on the placement and the only thing I heard him say was “smoke.” He doesn’t interact or talk any further than that. But that day ... it was like seeing a different person altogether. He was talking about his favourite ... he was talking about cars, I was actually in awe and I think that’s really going to stand out for me. It was like watching a different person altogether.

Participant 2, focus group 1

These moments challenged students’ assumptions about dementia and reframed how they understood residents’ capacity for expression.

Students consistently emphasized the importance of individualization, noting that the most meaningful responses occurred when the cues uploaded to the app genuinely reflected the residents’ own interests, history, and preferences. They described how individual photographs, music, and videos attached to meaningful memories were perceived to elicit stronger engagement and more noticeable changes in emotional expression or communication:

It’s personal for them so it’s meaningful for them. As you say, knitting whatever else, maybe not for that person.

Participant 6, focus group 2

Students learned that knowing such histories is not optional or “nice to have” (Participant 13, focus group 4) but has the potential to have therapeutic benefits. They described how, in their experience, specific memorabilia could “reach” someone who otherwise “backs off” (Participant 2, focus group 1), reinforcing the direct link between individualization and therapeutic impact. This prompted deeper reflection on how to sustain such moments of connection: “I think the question that stayed with me is how do we get that person, how do we get him to live in that space because it was such a beautiful space” (Participant 4, focus group 1).

Another aspect of learning involved reinterpreting behavior as communication of need rather than as a fixed symptom of dementia. Individual reminiscence and access to life story information helped students link behavior with unmet needs. There was evidence of clinical reasoning, where they connected knowledge of life story and preferences with behavioral presentation and emotional regulation:

Because before, shouting and behavioural issues and things that was going on I did not really know why. And the nurses did not have time to explain why whenever they were running round trying to keep everybody on top. Just seeing then that was just for company, he just wanted company, he just wanted to tell his life story, he just wanted to go over and over things.

Participant 8, focus group 2

Through these experiences, students learned that behavior is often an expression of loneliness, distress, or need for familiarity and comfort.

### Theme 2: Learning Through Connection

Students explained that using the app helped them form stronger, more meaningful connections with residents by shifting the focus from routine tasks to shared conversation. Several participants described how reminiscence created a foundation for trust and how connection can foster comfort, familiarity, and willingness to engage: “It builds more connections as well, which doesn’t always happen when someone goes into a nursing home” (Participant 3, focus group 1).

Students also reported that the app helped them develop skills in initiating meaningful conversations, deepening discussion, and creating opportunities for residents to express themselves. The process appeared to teach students how to structure conversations around personal histories rather than routine questions:

Do you know, I think the project actually helped get me asking the questions. Usually the resident would tell you about their life. It got them into maybe a deeper discussion about their life and then they could reminisce more about memories in the past.

Participant 12, focus group 4

Students consistently described gains in active listening, patience, and presence:

We love to talk and sometimes we need to learn how to listen to other people as well, just be quiet and be there and wait, not expect everything to go very quickly and be patient with the patient.

Participant 10, focus group 3

Students also described learning how to collaborate more effectively with families. The app positioned families as essential partners in gathering meaningful content and understanding the resident:

It’s really important to learn about that from family or whatever resources you can get. It helps you kind of respect that.

Participant 2, focus group 1

This strengthened students’ communication skills, particularly in their interactions with families. The project also enabled some students to recognize the value of engaging with relatives more routinely. By initiating these conversations, they gained deeper insight into the residents’ history, preferences, and needs, which supported more informed and person-centered care.

I would say communication, yeah, that’s improved my communication because I have to communicate with the daughter, I have to call her on the phone and the day she’s going to call and all that.

Participant 6, focus group 2

Students also became more aware of the complexities surrounding limited or fractured family involvement. For some, initiating contact highlighted emotional challenges, sensitivity, and the need for a respectful approach when family members were struggling or visited infrequently:

Well, the person ... is 47 ... So, I had found that quite sad, and then finding out a bit of his background to having a child. Kind of limited family. Maybe a visit once a week really. Not much more than that. I did find it a wee bit of a struggle communicating with the dad, not because the dad was making it difficult to communicate but I found the situation a wee bit difficult, but I learnt from that.

Participant 11, focus group 3

Equally, students recognized the relational benefits for families themselves, noting that reminiscence activities often strengthened connections between relatives and their loved one:

She was really coming through, and that was like really nice to see but then you could see the family sometimes tear up a wee bit ... they were really connecting with how their mum was before she got dementia.

Participant 1, focus group 1

Some students also identified intergenerational benefits, noting how reminiscence tools supported younger family members to connect with residents in ways that had previously been difficult. These observations helped students appreciate how technology mediated communication across age groups and facilitated shared interaction:

It’s maybe giving them something to focus on, that bridge or something, that connection between a grandmother who they couldn’t communicate but now there’s something there to help this communication. And technology as well, we all know kids love technology, so it works.

Participant 12, focus group 4

Students also expressed that although they initiated much of the reminiscence work, they hoped families would continue it, having seen its value for residents and relatives alike. They observed families taking ownership of the app and building on what the students had begun*:*

I put basic photographs and a couple of songs onto it, whereas the family, there’s three members of the family, they love it, they’re just gonna keep adding to it and adding their own personal photographs, if you like, and they’re starting to record voice notes onto that. It’s good for them, it’s really good for the family because they come in every day and different members of the family where they all have this collection now.

Participant 8, focus group 2

Some students discussed how the reminiscence process facilitated powerful reconnections between residents and people from their past. In one case, the student enabled renewed family involvement for a resident who had lived in the care home for more than 20 years:

Aye so ours, so she’s been in the care home now for over 20 years. Not a lot of family. I’ve never seen family there ... I was able to kind of reintroduce that kind of family connection again which was lovely .... It definitely had reconnected her with her family again. And they’re glad now they’re back in her life again after so long. I think maybe they thought she’s been in there years, she’s got dementia, there’s no point in going near her.

Participant 3, focus group 1

These moments helped students appreciate the emotional and relational rewards of person-centered care and to find genuine joy in facilitating meaningful reminiscence:

I think she was enjoying looking at the photos and it was really interesting for me to hear so much about her life and she travelled so extensively and hearing all about this was really interesting, so I really enjoying putting them all together onto the app.

Participant 9, focus group 3

Alongside these positive experiences, some students also expressed uncertainty and concern about the potential emotional impact of reminiscence. Students reflected on not wanting to cause any harm or distress to the resident, recognizing that not all memories are happy. They described actively balancing the emotional impact of reminiscence and the need to protect residents from potential distress.

And then there was one day she was a wee bit upset because she was at the nurses station saying that she wanted to go home so I was afraid was I upsetting her by bringing up family members again.

Participant 7, focus group 2

So, I suppose it goes back to that whole trauma, is there trauma that you are bringing up then but then is negative stimulation better than no stimulation? What is that?

Participant 4, focus group 1

And it is quite a sad case and then I did not want to probe too much.

Participant 12, focus group 4

Students recognized that meaningful engagement generates mutual reward for the resident, themselves, and the family and can improve residents’ emotional well-being, which reinforced the value of relationships.

### Theme 3: Growing as Person-Centered Practitioners Within the Realities of Care Home Practice

While previous themes describe changes in how students understood and connected with residents, this theme focuses on how they applied this learning in practice within the constraints of care home environments.

Students described how new insights influenced their practice, encouraging them to adopt a more strengths-based approach:

Dementia doesn’t have to be the end for the person. They still have a life, they still have interests, they still have things that will make their face light up, like their legs tapping to music and you know.

Participant 12, focus group 4

Through this, students were not only learning about residents but learning how to think differently in practice: to look beyond diagnosis, assume uniqueness, and adapt care rather than standardize it. These moments seemed to prompt students to look more closely at individual histories and to consider how personal background might shape current needs and experiences.

To be more person-centred and more holistic and just don’t look at them oh, they have dementia, they’re gonna have a bad day.

Participant 10, focus group 3

Students described how initial fears and uncertainties were gradually replaced with confidence as they spent time with residents. Early apprehension often stemmed from limited prior experience, preconceived expectations, or narratives inherited from staff, leaving some unsure of how to approach individuals or interpret behaviors:

I was not given a good outlook from the other nurses and things so, I did not really know how to approach, but then ... she was just an absolute lady.

Participant 5, focus group 1

Working closely with residents fostered a sense of curiosity that prompted students to look beyond routine interactions and explore what mattered to individuals. Curiosity encouraged them to investigate residents’ interests, make sense of subtle behavioral responses, and try out more individual approaches. This positioned students not only as learners but as emerging contributors to care. Several described using their developing insights to guide decisions, suggest activities, and influence how others engaged with residents, signaling early forms of leadership in person-centered practice.

As students grew more confident, they began to show initiative and demonstrate leadership abilities through creative problem-solving, advocating for residents, and seeking additional resources when needed:

I think it is just also the type of just always trying to dig deeper and try to maybe think outside the box as in music might not work for somebody but for somebody it might be taking them to the garden for the light than apps. I think it is just the patience and confidence to keep on digging.

Participant 7, focus group 2

Beyond demonstrating curiosity and initiative, some students described going above and beyond for residents in ways that reflected early leadership in person-centered practice. These actions were grounded in values of dignity, identity, and meaningful connection. One powerful example came from a student who noticed that a resident frequently spoke with pride and emotion about her past involvement in a local choir. Wanting to enhance the resident’s experience, the student explored this part of her history in a general way, without sharing personal information, to see whether any archival material or background information could be located:

So, I reached out. My wee lady talks away about she was in the choir in [redacted] and when she talks about it her eyes, and she travelled around the world and was singing in front of thousands of people and loved it ... so I contacted the [redacted] Choir.

Participant 3, focus group 1

Through this exploration, the student was able to source historical material and reconnect the resident with aspects of her earlier life:

They’ve sent me some of the music, some of the clippings and messages and I’ve been reading through it with her and she’s been loving it, it’s making her relive her days. There’s a picture of a lady that she shared a bedroom with when she was going travelling round the world and they shared the room and all together, and then she’s talking away.

Participant 3, focus group 1

This demonstrates how students began to enact person-centered values through advocacy, signaling a shift from participation in care toward active influence within the practice environment. Such initiative was sometimes recognized by staff, sparking wider interest in individual reminiscence: “So much so that my PA [Practice assessor] wanted more information, she wanted to see if it was possible for her to start doing a bit more” (Participant 9, focus group 3).

These positive encounters contributed to strong feelings of pride and job satisfaction. Students also reflected on themselves and the kind of practitioner they aspired to become:

There was some sort of rewarding feeling doing it ... A job satisfaction feeling.

Participant 1, focus group 1

These experiences also sharpened students’ awareness of the broader care environment. Reminiscence work highlighted how routine-driven care can overshadow residents’ individuality and emotional needs:

I think it shows a whole other side because going in as a student you sort of learning routines and tasks, but this brings to the forefront that they are human beings and seeing a whole side of them that you never would have ordinarily see.

Participant 6, focus group 2

Despite these positive developments, students consistently highlighted challenges in sustaining this approach within busy care environments. Students described becoming more aware of the organizational pressures that make meaningful and sustained engagement difficult including high workloads, competing demands, limited time, and task-driven routines:

I know it is so busy but sitting down and actually having more one to one conversations with the residents instead of just, I know it is so busy, you are just running about all day doing everything and it is like you do not actually have the time to sit and talk to them.

Participant 5, focus group 1

Students also observed how staff roles and organizational structures shaped what was possible. They felt that staff interest and prioritization played a crucial role in determining whether reminiscence work could be embedded and noted that such activities were often viewed as the responsibility of caregivers rather than nurses:

I think the carers are nearly more important than the nurses because they’re the ones that are actually with the residents and they know them 10 times better ... nurses are limited to what they do nearly like in a negative way, where it’s kind of like that’s not my job, that’s the carers.

Participant 5, focus group 1

Practical barriers also emerged around technology access and management support. Several students described difficulties with app setup, variable communication within teams, and inconsistent managerial responses:

We had a problem with the care home actually setting it up ... we went to management but that never really materialised.

Participant 7, focus group 2

The staff are changing on such a constant basis ... you had to explain it all over again and maybe they weren’t really listening.

Participant 3, focus group 1

At times, organizational culture actively constrained student engagement. Instances of being discouraged from using the iPad created uncertainty and inhibited initiative:

The manager came down and said we weren’t allowed to sit on the iPad ... after that if I got caught on it I felt I would get in trouble.

Participant 1, focus group 1

Students also recognized that their own academic responsibilities created competing priorities:

Yes, me too, in that week I had not touched the iPad because I was like well, I need to pass this year so I am going to focus on that.

Participant 8, focus group 2

Despite these pressures, students discussed how reminiscence and meaningful engagement are core elements of nursing care, not optional extras. They positioned relational connection as just as important as clinical skill:

But also some people go into nursing and they think it is all only about clinical skills but it is not, it is about sitting down and being with a person and making connections rather than just passing through, you know.

Participant 10, focus group 3

Students learned through direct observation. Their understanding of reminiscence shifted from theory to lived evidence as they saw nonverbal or minimally verbal residents speak, recall, or visibly relax. They emphasized that engagement in the project enabled them to integrate their theoretical learning into practice, turning abstract concepts into meaningful understanding:

Definitely. I think it just gave me a better insight in itself, I know we done learning and stuff here, I think when you are more hands on it just opened my eyes a lot more, so it did.

Participant 2, focus group 1

Like what I done before I obviously gained consent or anything was learn about what the app is and what Reminiscence means or what that is. So, in that sense, if somebody was asking me things I would actually understand. Whereas beforehand, you learn about it but actually, putting theory into practice, that is where I gained confidence.

Participant 6, focus group 2

For many students, these experiences prompted suggestions about how reminiscence could be embedded into the curriculum:

If it is there and something you have to do then you see the benefits of that, otherwise you can just trudge on through your daily routine and you do not see that.

Participant 10, focus group 3

These findings demonstrate how individual reminiscence supported students to develop confidence, communication skills, reflective awareness, and a clearer understanding of what person-centered care requires in practice. The work enabled them to begin integrating their learning into practice while also recognizing the cultural and structural factors that shape care delivery. Through this, students strengthened their commitment to relational, person-focused nursing within the realities of the care home environment.

## Discussion

### Principal Findings

The findings indicate that engaging with the InspireD reminiscence app during placement enabled students to move from task-focused interactions toward relational, person-centered ways of understanding and delivering care. Working with residents’ life histories created opportunities for meaningful connection with residents and families, challenged deficit-based assumptions about dementia, and supported the translation of person-centered values into everyday practice. Through this process, students appeared to develop not only competence but also a clearer understanding of what person-centered care looks like within the realities of care home practice. By examining how engagement with residents’ life stories during placement shaped students’ perceptions, reasoning, and actions, these findings offer empirical insight into how person-centered values can be translated from curriculum into practice. To the authors’ knowledge, this is the first study to explore this process using an individualized digital reminiscence approach within practice learning in care homes, thereby addressing an important contextual gap in the literature. However, it is not possible to attribute learning specifically to the app, as findings reflect the cumulative influence of prior education, placement experience, and engagement with residents.

There is limited empirical evidence describing how person-centered values in dementia care, taught within university curricula, are operationalized and enacted by students in real practice environments [3]. Education within university nursing programs involves more than the transmission of knowledge. Learning within these contexts should extend beyond the presentation of information to create learning experiences that support student nurses to internalize values, develop professional identity, and shape how care is understood and delivered [29-31]. Increasingly, this is achieved through learning experiences that are relational, experiential, and situated within practical contexts [3,32]. While students are expected to graduate with the values and attributes of person-centered practitioners, these are unlikely to develop through passive learning alone. Dewing [33] emphasizes the importance of active engagement, reflection, and shared learning in supporting transformation within practice cultures, a perspective that aligns with experiential learning theory, where learning is understood as an ongoing process integrating experience, reflection, and action.

Person-centeredness within the nursing curriculum is a values-based, humanizing philosophy that emphasizes supportive learning cultures, practitioner confidence, and authentic therapeutic relationships [34]. Educational approaches grounded in personhood are argued to be central to person-centered curricula, requiring higher education institutions to support learning experiences that encourage reflection on values, beliefs, and assumptions [35,36]. Such reflection is fundamental to the development of cultural humility, whereby learners critically examine how their perspectives shape interactions with others [37]. However, a recent review by Gonzalez-Moreno et al [38] highlights a lack of empirical studies that integrate values-based education in humanization with digital technology. They note that digital tools are more commonly framed in terms of efficiency or skills acquisition, rather than as relational resources capable of supporting values-based learning. The findings of our study suggest that when used purposefully, everyday digital tools such as the InspireD reminiscence app may offer opportunities for person-centered learning that support reflection, connection, and engagement with personhood. This study therefore provides empirical evidence that a digital reminiscence resource can function as a relational pedagogical mechanism for values-based learning within practice placements, extending existing work that has primarily positioned digital tools in terms of technical skill development or service efficiency. Future research could incorporate more structured implementation approaches, including documenting frequency and duration of app use, to better understand the relationship between level of engagement and learning outcomes. Combining qualitative insights with measures of exposure may help to clarify the mechanisms through which digital reminiscence supports experiential learning in practice settings. Nevertheless, student perspectives were captured at a single point in time within a specific context. Further comparative research is therefore needed to explore how digital reminiscence approaches differ from, or add to, nondigital methods, including consideration of feasibility, sustainability, and contextual fit within care environments.

Students’ learning was also shaped by the wider practice environment. These experiences reflect wider research on care home environments, which highlights the impact of organizational context on both care delivery and learning opportunities [39-41]. Supportive learning cultures are particularly important if person-centered values are to be embedded. This is well recognized, with several authors [42-44] emphasizing the influence of care home culture on learning experiences. These findings add to this body of work by highlighting the importance of openness to innovation as a cultural condition that shapes how new approaches are received, enacted, and sustained in practice. There is an increasing recognition of the need for openness to innovation and acceptance of new ways of working within care homes if novel interventions are to be adopted by staff and supported during student placements [45]. Supporting practice supervisors and care home staff to understand the educational purpose of individual reminiscence as well as the technology may therefore enhance the consistency and quality of student learning and represent an important area for practice development.

Additionally, an important point is raised around continuity of care. Where students introduce individualized reminiscence practices that appear to be of benefit, questions arise about the implications of how these practices are sustained in practice once the student placement ends. World Health Organization [46] guidance on digital interventions for health system strengthening emphasizes that when digital tools become embedded in care practices and relationships, their discontinuation may be experienced as a loss, particularly where they have informed care or supported relational connection. This highlights the ethical importance of considering sustainability from the outset. This is doubly important when considering a digital innovation. In this study, each student was provided with an iPad for use, with the device remaining in the care home following completion of the activity. While this facilitated access, it raises questions regarding responsibility for device management and upkeep. Although the InspireD reminiscence app includes backup features, the implications of device failure and ongoing device upkeep remain significant. Future research should consider the practical and economic implications of sustaining digital reminiscence, including device provision, maintenance, replacement, technical support, and the longer-term sustainability of these technology-facilitated reminiscence within care settings.

These findings contribute to wider discussions on the role of digital tools in health professions education [47-49]. Further work is also needed to examine the longer-term impact of individual digital reminiscence on students’ learning and professional development. Incorporating the perspectives of residents, families, and care home staff would support a more comprehensive understanding of how individual reminiscence is experienced across care environments. Extending this work to include other health care learners, alongside larger multisite studies, would help to explore the transferability of these findings and identify the conditions under which individual digital reminiscence can be meaningfully embedded within health care education and practice.

### Strengths and Limitations

This study provides in-depth qualitative insight into student nurses’ learning through personalized digital reminiscence in care home settings, addressing an underexplored area in nursing education. The flexible and practice-embedded nature of the intervention may be considered a limitation, as the extent and frequency of app use varied across participants and were not standardized or formally quantified. As a result, it is not possible to isolate the specific contribution of the app from other aspects of placement learning, including broader interaction with residents and families. However, this reflects the realities of care home practice and was central to understanding how digital reminiscence is integrated within everyday care. This study was conducted within a specific regional context and involved a relatively small sample, which may limit transferability to other settings. Participants were self-selecting student nurses who had chosen to take part in a university-supported project, and it is therefore possible that the sample was skewed toward students who were more motivated, confident, or positively disposed toward relational or innovative approaches to care. The use of focus groups to explore individual subjective experience may have limited the depth of some personal accounts, as one-to-one interviews can facilitate more detailed exploration. Although the group format enabled reflective dialogue and comparative discussion, it is possible that some participants moderated their views in the presence of peers. In addition, participants were aware that the study was associated with their educational institution, which may have increased the likelihood of socially desirable responses. Despite mitigation strategies, the involvement of researchers in the wider academic program may have influenced how freely some students expressed critical views. Data capture reflected student experiences at a single point in time, restricting insight into longer-term impacts on professional development and practice. Additionally, participating care homes were engaged in the My Home Life program, which may have fostered a culture of reflection and openness to innovation. Experiences of implementing the app may therefore differ in settings without similar organizational readiness. In addition, the study focused on student perspectives and did not include the views of residents, families, or care home staff, which would provide a more comprehensive understanding of how personalized reminiscence is experienced across the care environment. These limitations highlight important directions for future research, including longitudinal and multistakeholder studies. Despite these limitations, the study offers novel empirical insight into how person-centered values can be enacted through practice-based learning and highlights important contextual conditions for the implementation of digital reminiscence in care home education.

### Conclusions

These findings contribute to emerging literature on experiential learning in nursing education by exploring student nurses’ engagement with the InspireD reminiscence app in care home settings. The findings highlight how the app can support students to engage with personhood, develop empathy, and reflect on person-centered care in ways that extend beyond task-focused practice. Importantly, the study demonstrates that meaningful use of digital tools, rather than access alone, is central to their educational value. Students’ experiences also draw attention to the influence of organizational culture, learning environments, and staff engagement in shaping what can be sustained in practice. These findings suggest that the InspireD reminiscence app has potential as a structured learning activity within nursing education, particularly where learning cultures support reflection, innovation, and person-centered practice. By identifying the contextual and pedagogical conditions that enable person-centered values to be translated from curriculum into practice, this study offers a foundation for future longitudinal and multistakeholder research and for the development of sustainable, values-based digital learning approaches across health professions education.

## Abbreviations

COREQ: Consolidated Criteria for Reporting Qualitative Research
iCHECK-DH: Guidelines and Checklist for the Reporting on Digital Health Implementations

## Data Availability

Anonymized data may be available from the corresponding author on reasonable request.
